# Supplementary material for: Substance use patterns among individuals with problematic pornography use: A scoping review
Source: PLOS Glob Public Health. 2025 Nov 12;5(11):e0004946. doi: 10.1371/journal.pgph.0004946 (PMC12611143; doi:10.1371/journal.pgph.0004946)
Supplement: S1 File — (DOCX) [file pgph.0004946.s001.docx]

**S1 Appendix: List of Eligibility Questions**

1. Inclusion criteria:
   1. The article must:
      1. Include any [problematic/chronic/uncontrolled] pornography use, even if it is the not primary objective of the paper.
      2. Include any substance use as defined by the Centers for Disease and Control
      3. Include an outcome measure that examines the relationship between pornography use and substance use.
      4. Be peer-reviewed
      5. Be available in English
      6. Involve human subjects
