# Supplementary material for: Substance use patterns among individuals with problematic pornography use: A scoping review
Source: PLOS Glob Public Health. 2025 Nov 12;5(11):e0004946. doi: 10.1371/journal.pgph.0004946 (PMC12611143; doi:10.1371/journal.pgph.0004946)
Supplement: S2 File — (DOCX) [file pgph.0004946.s002.docx]

**S2 Appendix: Search Terms**

('drug dependence'/exp OR 'illicit drug'/exp OR 'alcoholism'/exp OR 'substance related disorder*' OR 'substance use disorder*' OR 'substance abuse' OR 'substance dependenc*' OR 'substance addiction*' OR 'chemical dependenc*' OR 'drug use disorder*' OR 'drug abuse*' OR 'drug misuse*' OR 'drug dependenc*' OR 'drug addict*' OR 'illicit drug use' OR 'illicit drug abuse' OR alcoholism OR 'binge drinking' OR 'marijuana use' OR 'marijuana abuse' OR 'cannabis use' OR 'cannabis abuse' OR 'tobacco use*' OR smoking OR cigarette* OR vaping OR 'e cigarette*' OR 'nicotine addiction*' OR 'narcotic related disorder*' OR 'narcotic abuse*' OR 'narcotic addiction*' OR 'opioid addiction*' OR 'opioid use' OR 'opioid abuse*') AND ('sexual addiction'/exp OR 'compulsive sexual behavior disorder*' OR csbd OR 'pornography use disorder*' OR 'problematic pornography use' OR 'pornography addict*' OR 'compulsive sexual behavior*' OR 'internet porn*' OR 'porn addict*' OR (('internet'/exp OR internet* OR online) AND ('erotica'/exp OR 'pornography'/exp OR erotica OR porn*)))
