# Supplementary material for: Substance use patterns among individuals with problematic pornography use: A scoping review
Source: PLOS Glob Public Health. 2025 Nov 12;5(11):e0004946. doi: 10.1371/journal.pgph.0004946 (PMC12611143; doi:10.1371/journal.pgph.0004946)
Supplement: Attachment — Submitted filename: PGPH-D-25-01648.pdf [file pgph.0004946.s004.pdf]

Summary

This manuscript offers a timely and well-executed scoping review examining the intersection between problematic pornography use (PPU) and substance use—an area of growing interest but limited empirical exploration. Drawing on eight cross-sectional studies, the authors map the emerging evidence on co-occurring substance use among individuals with PPU and underscore the need for standardized definitions and more rigorous research in this domain. The review is clearly written, methodologically sound, and adheres to PRISMA-ScR guidelines. It makes a valuable contribution to the behavioral and global health literature, especially considering the stigma and complexity surrounding both PPU and substance use.

Thank you for the opportunity to review this important and thought-provoking manuscript. Below, I outline key strengths of the study, along with a few constructive suggestions to support the authors in refining their final submission. I look forward to seeing this work published and advancing the conversation in this critical area of global health.

Strengths

1. **Timeliness and Relevance:** The study addresses an important and often stigmatized topic that has received little empirical attention, particularly the co-occurrence of PPU and substance use.
2. **Clear Objectives:** The aims of the review are well defined, and the rationale for exploring this intersection is thoroughly explained, particularly in relation to public health implications.
3. **Rigorous Methodology:** The authors followed a structured and transparent scoping review process, including PRISMA-ScR guidelines and OSF registration, enhancing reproducibility.
4. **Comprehensive Database Search:** The search strategy spans multiple major databases and includes clearly defined inclusion/exclusion criteria, contributing to the methodological robustness.
5. **Well-Structured Results:** The synthesis of findings is clearly presented through well-organized tables and a detailed narrative summary that makes it accessible to both clinical and academic audiences.
6. **Practical Implications:** The discussion section meaningfully interprets the findings and offers important reflections on the need for standardized tools and longitudinal research.

Areas for Improvement

While the manuscript is strong overall, the following minor revisions are recommended to enhance clarity, rigor, and scholarly impact:

1. Terminology Consistency and Justification

- The manuscript refers to PPU, pornography addiction, PUD, and chronic pornography use somewhat interchangeably. While the authors acknowledge the lack of standard definitions, they should:

    - Provide a clearer justification for using PPU as the primary term throughout.
    - Consider limiting the use of potentially stigmatizing terms like "pornography addiction" or at least contextualize their use based on the literature.

2. Quality Appraisal of Included Studies

- Although scoping reviews typically do not include a formal risk of bias assessment, a brief comment on the overall quality or limitations of the included studies (e.g., small sample sizes, self-reported measures, cross-sectional design) would enhance the critical interpretation of findings.

3. Clarify Operationalization of Substance Use

- The classification and measurement of substance use vary across studies (e.g., AUDIT, BASIS-24, chemsex definitions), and while Table 2 is helpful, the main text would benefit from a clearer synthesis of how these differences may have influenced comparability or findings.

4. Expand on Implications for Policy and Practice

- The manuscript briefly touches on integrated interventions and stigma, but could go further in discussing:
    - How health professionals and public health systems might respond to overlapping PPU and substance use.
    - Potential barriers to screening or treatment.
    - Implications for adolescent health and digital literacy programs.

5. Minor Edits for Clarity

- A light copy edit is recommended to correct minor grammatical issues and improve sentence clarity in several sections (e.g., Abstract and Discussion).
- Example: The sentence "PPU operates similarly to substance-based addictions by stimulating similar pathways…" could be revised to avoid redundancy.

Conclusion and Recommendation

This manuscript makes a meaningful contribution to public health literature by mapping the sparse but emerging evidence on substance use in the context of PPU. The study is well-conducted, clearly written, and addresses a stigmatized and underexplored topic with sensitivity and academic rigor.

I recommend acceptance pending minor revisions to improve consistency in terminology, strengthen interpretive clarity, and expand briefly on practical implications for intervention and policy development.
