# Supplementary material for: Substance use patterns among individuals with problematic pornography use: A scoping review
Source: PLOS Glob Public Health. 2025 Nov 12;5(11):e0004946. doi: 10.1371/journal.pgph.0004946 (PMC12611143; doi:10.1371/journal.pgph.0004946)
Supplement: Attachment — Submitted filename: Response to Reviewers.docx [file pgph.0004946.s006.docx]

August 15^th^, 2025

Susmita Chandramouleeshwaran
Academic Editor
PLOS Global Public Health

Dear Dr. Chandramouleeshwaran,

Thank you for the opportunity to revise and resubmit our manuscript entitled, “Substance Use Patterns Among Individuals with Problematic Pornography Use: A Scoping Review” (**PGPH-D-25-01648**). We have addressed all of the editors and reviewers concerns and modified our manuscript accordingly.

We look forward to your decision.

Best wishes,

Dr. Cerina Dubois

**Comments to the Author:**

**C1. Please amend your online Financial Disclosure statement. If you did not receive any funding for this study, please simply state: “The authors received no specific funding for this work.”**

R1. We have revised the statement to reflect the above. (please see page 18 of manuscript)

**C2. Please update your online Competing Interests statement. If you have no competing interests to declare, please state: “The authors have declared that no competing interests exist.”**

R2. We have revised the statement to reflect the above. (please see page 18 of manuscript)

**C3. In the online submission form, you indicated that “Data extraction supplementary tables can be accessed by request to corresponding author.”.**

**All PLOS journals now require all data underlying the findings described in their manuscript to be freely available to other researchers, either 
a) In a public repository, 
b) Within the manuscript itself, or 
c) Uploaded as supplementary information.**

R3. This comment was also echoed by one of the reviewers. All of our findings will be freely available to other researchers in the Open Science Framework repository. We have updated the manuscript to reflect this. (please see page 18 of manuscript)

**C4. Please provide separate figure files in .tif or .eps format only and ensure that all files are under our size limit of 10MB. You may leave the embedded figures in the manuscript.**

**For more information about how to convert your figure files please see our guidelines:** [**https://journals.plos.org/globalpublichealth/s/figures**](https://journals.plos.org/globalpublichealth/s/figures)

R4. We have provided separate figure files in .tif formats and have left them embedded in the manuscript.

**C5. If the reviewer comments include a recommendation to cite specific previously published works, please review and evaluate these publications to determine whether they are relevant and should be cited. There is no requirement to cite these works unless the editor has indicated otherwise.**

R4. The other reviewers have not required this.

**C6. Please review your reference list to ensure that it is complete and correct. If you have cited papers that have been retracted, please include the rationale for doing so in the manuscript text, or remove these references and replace them with relevant current references. Any changes to the reference list should be mentioned in the rebuttal letter that accompanies your revised manuscript. If you need to cite a retracted article, indicate the article’s retracted status in the References list and also include a citation and full reference for the retraction notice.**

R6. Certainly. We have ensured to check our reference list.

____
**Reviewers' comments:**

**Reviewer 1:**

**C7. The** [**PLOS Data policy**](https://nam02.safelinks.protection.outlook.com/?url=https%3A%2F%2Ftrack.editorialmanager.com%2FCL0%2Fhttps%3A%252F%252Fjournals.plos.org%252Fglobalpublichealth%252Fs%252Fdata-availability%2F1%2F010f0198a77c0345-eb401937-f335-4a34-9ca1-7a26d740cee2-000000%2Fy2Xwli9PSh__Uc7kMWWGXRr9VYwQRzvZte5eTl0aSSc%3D224&data=05%7C02%7Ccdubois6%40jh.edu%7C40521ebb665a4bf25be908dddb0454da%7C9fa4f438b1e6473b803f86f8aedf0dec%7C0%7C0%7C638907533868529719%7CUnknown%7CTWFpbGZsb3d8eyJFbXB0eU1hcGkiOnRydWUsIlYiOiIwLjAuMDAwMCIsIlAiOiJXaW4zMiIsIkFOIjoiTWFpbCIsIldUIjoyfQ%3D%3D%7C0%7C%7C%7C&sdata=ONHWwH7c8MBOW2zXZTQc0mlVWhEztCRurMXcPsDq88w%3D&reserved=0) **requires authors to make all data underlying the findings described in their manuscript fully available without restriction, with rare exception. The data should be provided as part of the manuscript or its supporting information, or deposited to a public repository. For example, in addition to summary statistics, the data points behind means, medians and variance measures should be available. If there are restrictions on publicly sharing data—e.g. participant privacy or use of data from a third party—those must be specified.**

**Reviewer #1: No**

R8. We have amended this as reflected above in R3 – to ensure that our findings can be freely accessible to all researchers in the Open Science Framework registration link.

**C8. Terminology Consistency and Justification**

**The manuscript refers to PPU, pornography addiction, PUD, and chronic pornography use somewhat interchangeably. While the authors acknowledge the lack of standard definitions, they should: Provide a clearer justification for using PPU as the primary term throughout. Consider limiting the use of potentially stigmatizing terms like “pornography addiction” or at least contextualize their use based on the literature.**

R8. We agree with this reviewer that a clearer justification and clarity surrounding the term, PPU, is needed. We agree that “pornography addiction” has not been officially operationalized as a clinical addiction by the DSM-5 and thus, we have removed this when we are explaining PPU in our Discussion. However, please note that in the majority of the 8 cross-sectional studies, the researchers still utilized the term “pornography addiction,” hence, why we did present this term in the Results when explaining the associations.

(Page 7; paragraph 2): “The review is not an examination pornography addiction as this is not a clinically recognized disorder/addiction in the DSM-5. However, it is important to note that our findings may still utilize this term, ‘pornography addiction’; and for the purposes of our scoping review, we will include that under PPU.”

**C9. Quality Appraisal of Included Studies**

• **Although scoping reviews typically do not include a formal risk of bias assessment, a brief comment on the overall quality or limitations of the included studies (e.g., small sample sizes, self-reported measures, cross-sectional design) would enhance the critical interpretation of findings.**

R9. Certainly. We have added more under the “Limitations” section of our manuscript.

(Page 17-18): “Specific to overall quality, the review only included 8 cross-sectional studies, which were reliant on self-reported measures. This may introduce recall bias and non-response bias. It is also likely that the study results may not be generalizable to individuals outside of the specific cohort. Furthermore, stigma surrounding pornography use may lead to underreporting due to individuals being more reluctant to disclose their usage due to fear of judgement (resulting in biased prevalence estimates). The underreporting can introduce confounding as those who report use may differ systematically from those who do not (in terms of their substance use outcomes). Hence, these differences may have underestimated true associations between pornography use and substance use outcomes.”

**C10. Clarify Operationalization of Substance Use**

**The classification and measurement of substance use vary across studies (e.g., AUDIT, BASIS-24, chemsex definitions), and while Table 2 is helpful, the main text would benefit from a clearer synthesis of how these differences may have influenced comparability or findings.**

R10. We want to clarify that AUDIT, BASIS-24, chemsex measures were all different because they measure different substance use categories (e.g., alcohol vs illicit drugs vs cannabis vs tobacco). The primary focus of our scoping review is to emphasize that there is no standard operationalized tool for measuring PPU (not substance use). We are unsure how to incorporate how this would have influenced comparability in our results as we are not comparing tools used within substance use types. We do agree that this is an important point and we are open to suggestions on how to tie this into our Discussion.

**C12. Expand on Implications for Policy and Practice**

**The manuscript briefly touches on integrated interventions and stigma, but could go**

**further in discussing:**

**◦ How health professionals and public health systems might respond to overlapping**

**PPU and substance use.**

**◦ Potential barriers to screening or treatment.**

**◦ Implications for adolescent health and digital literacy programs.**

R12. Thank you for the above suggestions. We do agree that we did not go in-depth on the stigma-aspect of PPU. Hence, we have added the following sentences to the research implications.

(Page 17-18): “Lastly, it is important to emphasize the stigma surrounding pornography use and how it remains a significant barrier to open dialogue, research, and clinical engagement.(25) Differences in culture, religion, and moral norms may often frame pornography consumption as deviant or shameful, which may lead individuals to conceal this behavior. This stigma not only contributes to underreporting in self-reported surveys, but may also affect help-seeking behavior, meaning those experiencing negative symptoms of PPU being reluctant to access support due to fear of judgement.(26) Consequently, this stigma can ultimately impact screening or receiving appropriate treatment from medical care professionals. From the health professional side, stigma may also impact their response to diagnosis, treatment planning, and appropriate referral pathways. Without a clear understanding of how PPU co-occurs with substance use, this may lead to fragmented treatment approaches that address substance use while missing appropriate interventions for PPU.”

**C13. Minor Edits for Clarity**

**• A light copy edit is recommended to correct minor grammatical issues and improve**

**sentence clarity in several sections (e.g., Abstract and Discussion).**

**• Example: The sentence “PPU operates similarly to substance-based addictions by**

**stimulating similar pathways…” could be revised to avoid redundancy.**

R13. We have read through the manuscript for areas of clarity.
